# Detecting Disease Outbreaks in Mass Gatherings Using Internet Data

**DOI:** 10.2196/jmir.3156

**Published:** 2014-06-18

**Authors:** Elad Yom-Tov, Diana Borsa, Ingemar J Cox, Rachel A McKendry

**Affiliations:** ^1^Microsoft Research IsraelHerzeliaIsrael; ^2^Centre of Computational Statistics and Machine Learning (CSML)Department of Computer ScienceUniversity College London, University of LondonLondonUnited Kingdom; ^3^Copenhagen UniversityDepartment of Computer ScienceCopenhagenDenmark; ^4^University College London, University of LondonDepartment of Computer ScienceLondonUnited Kingdom; ^5^University College London, University of LondonLondon Centre for Nanotechnology and Division of MedicineLondonUnited Kingdom

**Keywords:** mass gatherings, infodemiology, infectious disease, information retrieval, data mining

## Abstract

**Background:**

Mass gatherings, such as music festivals and religious events, pose a health care challenge because of the risk of transmission of communicable diseases. This is exacerbated by the fact that participants disperse soon after the gathering, potentially spreading disease within their communities. The dispersion of participants also poses a challenge for traditional surveillance methods. The ubiquitous use of the Internet may enable the detection of disease outbreaks through analysis of data generated by users during events and shortly thereafter.

**Objective:**

The intent of the study was to develop algorithms that can alert to possible outbreaks of communicable diseases from Internet data, specifically Twitter and search engine queries.

**Methods:**

We extracted all Twitter postings and queries made to the Bing search engine by users who repeatedly mentioned one of nine major music festivals held in the United Kingdom and one religious event (the Hajj in Mecca) during 2012, for a period of 30 days and after each festival. We analyzed these data using three methods, two of which compared words associated with disease symptoms before and after the time of the festival, and one that compared the frequency of these words with those of other users in the United Kingdom in the days following the festivals.

**Results:**

The data comprised, on average, 7.5 million tweets made by 12,163 users, and 32,143 queries made by 1756 users from each festival. Our methods indicated the statistically significant appearance of a disease symptom in two of the nine festivals. For example, cough was detected at higher than expected levels following the Wakestock festival. Statistically significant agreement (chi-square test, *P*<.01) between methods and across data sources was found where a statistically significant symptom was detected. Anecdotal evidence suggests that symptoms detected are indeed indicative of a disease that some users attributed to being at the festival.

**Conclusions:**

Our work shows the feasibility of creating a public health surveillance system for mass gatherings based on Internet data. The use of multiple data sources and analysis methods was found to be advantageous for rejecting false positives. Further studies are required in order to validate our findings with data from public health authorities.

## Introduction

### Background

Historically, infectious diseases have devastated societies. Examples include the “Black Death” bubonic plague of the 14th century in which between 30-40% of Europe’s population is estimated to have died [1], and the influenza epidemic of 1918-1920, in which as many as 50 million are estimated to have died [2]. Despite very significant advances in medicine, infectious diseases remain potentially very serious threats to society. For example, a pandemic influenza is rated as the greatest national risk on the UK government risk register [3]. An estimated 35.3 million people are HIV-infected [4], drug-resistant Methicillin-resistant *Staphylococcus aureus* (MRSA) is a major public health concern [5], about 2 million cases of cancer are caused by infections each year [6], and infection is a major source of morbidity in primary care [7]. Moreover, emerging new infections, such as H1N1 influenza, can cause pandemics, spreading rapidly and unpredictably. Early diagnostics play a crucial role in prevention, treatment, and care but most tests require samples to be sent to specialist laboratories leading to inherent delays between tests, results, and clinical interventions. Public health intervention may be further delayed by the time lag of 1-2 weeks associated with retrospective surveillance. There are increasing national and international drivers to dramatically improve our capacity to rapidly respond to infectious diseases by widening access to tests in community settings and drive innovative real-time surveillance

Protection against infectious diseases includes the development of new medicines, vaccination programs, improved hygiene, and promotion of behavioral modifications. While together these efforts may reduce the risk of infectious diseases, the risk cannot be eliminated. Consequently, infectious disease surveillance networks at national and international levels have been established. The purpose of public health surveillance networks is to provide “Ongoing systematic collection, analysis, interpretation and dissemination of data regarding a health-related event for use in public health action to reduce morbidity and mortality and to improve health” [8].

The most reliable sources of data for public health surveillance networks are confirmed diagnoses of diseases. Unfortunately, confirming a diagnosis may take days or weeks, due to a variety of delays including (1) time to ship a patient sample to a testing laboratory, (2) time to perform the test, and (3) time to report the results.

Delays in identifying the onset of an infectious epidemic result in delayed responses, which can significantly exacerbate the impact of the epidemic on a society. Consequently, there is strong interest in reducing delays. One way to accomplish this is through syndromic surveillance, which emphasizes “the use of near ‘real-time’ data and automated tools to detect and characterize unusual activity for further public health investigation” [9]. There is a range of pre-diagnostic data that can and has been used, including clinical data such as nurse advice line activity, school nurse visits, poison control center data, EMS records, emergency department visits, outpatient records, laboratory/radiology orders and results, prescription medication sales, and electronic health records, and non-clinical data such as over-the-counter (OTC) medications, work and school absenteeism records, ambulance dispatch data, zoonotic surveillance data (eg, dead birds from West Nile virus activity), health-related Web searches, and other data from online social networks.

The use of syndromic surveillance systems dates back to at least 1977, when Welliver et al [10] reported the use of OTC medication sales in Los Angeles. The early 2000s saw renewed interest in syndromic surveillance as a result of a US Defense Advanced Research Projects Agency (DARPA) initiative called ENCOMPASS (ENhanced COnsequence Management Planning And Support System) to provide an early warning system to protect against bioterrorism. As early as 2001, it was suggested to use query logs associated with health care websites as one form of syndromic data [11]. The advantage of online data sources is that the data collection is usually straightforward and very timely, that is, the lag between data creation, collection, and analysis can be very short (possibly seconds). We are therefore interested in online syndromic surveillance, which is discussed in more detail in the next section.

The World Health Organization (WHO) states that “an organized or unplanned event can be classified as a mass gathering if the number of people attending is sufficient to strain the planning and response resources of the community, state, or nation hosting the event” [12]. Examples of mass gatherings include very large religious gatherings such as the Hajj (approximately 2 million people) and the Hindu Kumbh Mela (estimated at 80-100 million people), large international sporting events such as the Olympics, and national music festivals such as Glastonbury in the United Kingdom. Mass gatherings have been sources for the spread of infectious diseases. The spread of cholera from a well in Mecca was documented as far back as 1883 [13]. More recently, during the 1992 Glastonbury music festival attended by 70,000 people in the United Kingdom, 72 cases of Campylobacter infection were reported due to drinking unpasteurized milk [14]. In 2009, [15] reported an outbreak of H1N1 influenza at the Rock Werchter festival in Belgium. Also in 2009, [16] reported outbreaks of H1N1 influenza at a sports event and at a music festival, called EXIT, where 62 confirmed cases were identified. In the same year, a further case was reported at a music festival in Hungary [17]. The issue of mass gatherings, medicine, and global health security was the subject of a series of reports in *The Lancet* in 2012.

In the next section, we provide a discussion of prior work on syndromic surveillance based on online social networks and search engine query logs.

### Related Work

In 2001, Wagner et al [11] first suggested the utility of query terms to detect infectious diseases. In particular, they presented data on the number of queries to a health website (WebMD) using words such as “cold” and “flu”. Though no quantitative assessment was provided, qualitatively a correlation is visible between the query frequency and measures of infectious disease. A related quantitative analysis was documented in subsequent work [18], which took “the weekly counts of the number of accesses of selected influenza-related articles on the Healthlink website and measured their correlation with traditional influenza surveillance data from the Centers for Disease Control and Prevention (CDC)”. The results showed a clear correlation; however, interestingly, the Web log data was no more timely than that of the CDC, that is, the Web log data did not allow an influenza outbreak to be detected any sooner than with traditional surveillance methods.

Later, Eysenbach [19] used information from Google’s AdSense to indirectly estimate the number of queries for particular search terms that contained keywords related to influenza. Specifically, Eysenbach reported correlations between the “number of clicks on a keyword-triggered influenza link” and traditional measures such as (1) the number of lab tests, and (2) the number of positive lab test results (cases). Pearson correlation scores of between .85 and .91 are reported. Interestingly, the higher correlation score was obtained when correlating with the number of cases reported for the next week, indicating the Web-based information was more timely.

A number of systems have been developed to gather and analyze unstructured information that is openly available on the Web. The earliest example of this is Global Public Health Intelligence Network (GPHIN) developed by the Canadian government and the WHO [20]. A number of systems have subsequently been deployed, including BioCaster [21,22], EpiSPIDER [23], and HealthMap [24,25]. Comparisons of these various systems can be found in [26,27].

Interest in Web-based surveillance increased significantly with the publication by Polgreen et al [28] and Ginsberg et al [29] of relationships between query search terms and influenza-like illness (ILI) based on Yahoo and Google search logs, respectively. Polgreen et al showed that it was possible to estimate the percentage of positive cultures for influenza and the deaths attributable to pneumonia and influenza in the United States, and to do so several weeks ahead of actual culture results. Ginsberg et al reported similar findings. A further contribution of [29] was to automatically determine the best set of query search terms that correlate with CDC estimates. The work by Ginsberg et al has subsequently been developed as Google Flu Trends and its more generic service, Google Trends [30].

A large body of research has since been developed that utilizes data from online social network or query logs to infer health information. This includes work on mining blog posts that mention influenza. For example, Corley et al [31,32] describe collecting blogs from a variety of sources and looking for the frequency of occurrence of keywords such as “influenza”. After normalization, they reported Pearson correlation scores of .77 and .55 for two datasets with corresponding ILI reports from the CDC (CDC ILINet reports). This work also discusses the possibility of identifying relevant online communities and developing associated targeted intervention strategies.

The analysis of microblogging data from Twitter for health purposes has recently received attention [33-40]. Inspired by the approach in Ginsberg et al [29], Cullota et al [35] applies a similar approach to Twitter data revealing the benefits of having longer, more complete messages as opposed to unstructured search query entries. This allows for simpler classification algorithms that can also filter out many of the erroneous messages that typically occur and would sometimes overwhelm the classifier predictions [38]. Lampos and Cristianini [33,34] performed an analysis of tracking influenza rates throughout the United Kingdom. Their major contribution to the existing regression-based models was proposing a new automatic way of selecting the keywords used by the classifier. These were learned from a large pool of candidates extracted from Web articles related to influenza, imposing a scarcity constraint via an L1 norm penalty in the least squares prediction error. This method yielded a correlation of 97% with respect to the reported influenza rates. Unfortunately, the proposed way of automatically building the vocabulary is based solely on correlation and sometimes produces terms that, although highly correlated with the flu trends, may not make good candidates to track for future predictions: for instance, automatically selected keywords “phone”, “nation”, or “mention” might not be good indicators of the presence of ILI conditions.

## Methods

### Data

We examined 10 events, nine of which were in the United Kingdom and one (the annual Hajj in Mecca) that had significant participation from people in the United Kingdom. All events took place in the second half of 2012.

We extracted two datasets for each event, one from the entire set of Twitter users and the other from that of the Microsoft Bing search engine. The population of Twitter users relevant to an event was defined as any user who mentioned a hashtag associated with an event at least twice between 30 days before and 30 days after the event. We refer to the relevant users as the target population. We also identified a population of users who could be used as a reference population (see Analysis Algorithms below) for each event by randomly sampling 1% of users who did not mention the event in their Twitter messages, but had the United Kingdom listed as their location in their profile. It comprised 345,849 users over the entire study period. For each Twitter message, we extracted an anonymized user identifier, the date and time of the message, and its text.

We followed a similar methodology for detecting relevant users according to queries made on the Bing search engine by users who agreed to share their queries, and marked as relevant any user who mentioned an event at least twice in their queries. For each query made by the relevant users, we extracted the query text, time and date, and an anonymized user identifier. In order to maintain user privacy, data were first anonymized by hashing, before the investigators had access to them. They were then aggregated prior to analysis and no individual-level user datum was examined by the experimenters.

On average, we identified approximately 14,000 Twitter users and 5650 Bing users. The list of events and basic statistics concerning the events are shown in Table 1, including the number of Twitter users who mentioned the event more than twice, the number of tweets that mentioned each event, the number of users who queried for each event, and the number of queries.

We extracted all queries and Twitter messages for the relevant users from 30 days before an event until 30 days after it. The queries and messages were stemmed using a Porter stemmer [41]. We then marked each query and Twitter message as to whether it contained one or more words or phrases describing medical symptoms given in a list of 195 medical symptoms and 457 corresponding synonyms described in Yom-Tov and Gabrilovich [42]. This list of terms was derived from a set of terms in *International Statistical Classification of Diseases and Related Health Problems, 10th Revision (ICD-10),* expanded to include ways in which non-specialist people frequently refer to the medical terms. The expansion is based on terms that people use in order to reach the Wikipedia page referring to a medical symptom and the terms frequently associated with it in Web documents. A complete explanation of how the list was constructed can be found in Yom-Tov and Gabrilovich [42].

A table listing the number of tweets that contained each of the symptom words or their synonyms in each of the festivals analyzed is provided in Multimedia Appendix 1.

**Table 1:** List of analyzed events and statistics.

Event	Dates	Capacity^a^	Twitter		Bing	
			Number of users	Number of festival mentions	Number of users	Number of festival queries
Wakestock	6-8 July	10,000	3878	12,180	1177	3750
Wireless Festival	6-8 July	50,000	23,105	191,762	2309	6909
T in the Park	6-8 July	85,000	24,746	175,881	11,899	44,416
V Festival	17-19 August	90,000	22,018	92,722	14,704	50,796
Bestival	6-9 September	30,000	13,359	104,550	6715	23,330
Creamfields	24-26 August	80,000	21,703	191,663	5533	19,071
Hajj	24-27 October	3,161,573	17,473	129,137	3402	13,892
Isle of Wight Festival	22-24 June	60,000	6276	1398	4400	14,222
Download Festival	8-10 June	120,000	9360	1497	4598	17,267
RockNess	8-10 June	35,000	12,935	1068	1764	6266
Median		70,000	15,416	98,636	4499	15,744

^a^Capacity information from Wikifestivals and Wikipedia websites.

### Analysis Algorithms

#### Overview

We analyzed each dataset using three methods, described below. Briefly, Method 1 tests how well the probability of a word occurring as a function of time fits a lognormal distribution with variance between 1.2 and 1.5, since this is the epidemiological distribution predicted in [43] for spread of infectious disease. Method 2 compares the number of times a symptom was mentioned before and after the date of an event, and uses a statistical test based on the False Discovery Rate (FDR) to determine significance. Method 3 computes the likelihood that symptoms would be measured at an observed frequency in a target population compared to what would be expected by chance. All three methods are described in detail below.

#### Method 1: Comparison to Background With Epidemiological Profile

Let *P* ^*T*^_*i*_ *(w,t)* be the probability that the *i*-th word will appear in the target population on day *t*, where, in our data *t*∈[−30,−29,...,29,30]. Similarly, we denote *P* ^*R*^_*i*_ *(w,t)* as the same probability in the reference population, that is, in a population that is disjointed from the target population, but is located in a similar geographic area.

We assume that if there is an epidemic of an infectious disease in the population, users mention its symptoms in their text (eg, Twitter messages). In that case, a word *P* ^*T*^_*i*_ *(w,t)* describing a symptom of the disease should follow the appearance profile of such a disease, which takes into account its incubation period. This profile should fit a lognormal distribution with a variance of between 1.2 and 1.5 [43].

Thus, for each of the symptom words, we compute its probability over time and normalize this by the same probability for the reference population, in order to exclude diseases that are unrelated to the event. Therefore, for each symptom word (and its synonyms), we compute a score given by *P* ^*T*^_*i*_ *(w,t)/ P* ^*R*^_*i*_ *(w,t)*, and fit to it a lognormal distribution with a center that varies from the first day of the event and until 14 days later. The day on which the best fit is found (in the least squares sense) is chosen to represent the distribution of this word.

In order to ascertain if the fit of the distribution is statistically significant, we employ the FDR procedure [44] and conduct the same procedure for a random set of 1950 non-symptom words (10 times larger than the symptom list) and display a symptom only if its fit to the lognormal distribution is greater than would be expected at an FDR of 1%.

This method should work well if there is a large enough target population to generate information pertaining to the epidemic and should enable not only the identification of the outbreak but also its temporal profile.

#### Method 2: Comparison to Background and Time

Here, we follow Yom-Tov and Gabrilovich [42] and construct a 2×2 contingency table that measures the number of times a symptom was mentioned before and after the date of the event (see Table 2 for an example), for either the target or reference population. Each symptom is then scored according to the chi-square score computed from the table.

A threshold for statistical significance is computed using FDR [44] with a random set of non-symptom words. We report symptoms with a chi-square score higher than that expected at an FDR of 1%.

**Table 2:** The 2x2 contingency table for computing the chi-square score of Method 2.

Number of times that the user mentioned/queried for the symptom or its synonym	User queried for or tweeted about the festival?	
	No	Yes
Before Day 0	N_11_	N_12_
After Day 0	N_21_	N_22_

#### Method 3: What’s Strange About Recent Events

Following the approach in [45] (What’s Strange About Recent Events [WSARE]), for each day after the mass gathering, *t*∈[1,⋯,30], we compute a one-term rule score for each symptom in our vocabulary. The score is computed using a hypothesis test in which the null hypothesis is the independence between history records and current day counts. We apply the Fisher’s exact test on a 2×2 contingency table, as shown in Table 3, made out of the current day’s symptom count and the number of times the symptom was mentioned in the time prior to the festivals.

The test generates a *P* value, given by *P(x=k)=C(K, k)C(N-K, n-k)/C(N, n)*, with *C(n, k)* being the binomial coefficient (“n choose k”) – *C(n,k)=n!/k!(n-k)!* and where *k* is the number of tweets containing the keyword *w*_*i*_ today, *K* is the number of times the keyword *w*_*i*_ was mentioned in the period before the festival, *n* is the number of tweets today, and *N* is the number of tweets in the period before the festival.

Since we are computing a score for each day, we consider as baseline the corresponding weekdays in the 30-day time window (ie, if the current day is Tuesday, we will look back to all Tuesdays in the time before the mass gathering and take that as our history baseline). This is done primarily to eliminate false detection due to periodic weekly trends in Twitter postings.

**Table 3:** The 2×2 contingency table (rule *w*_*i*_=1: tweet contains keyword *w*_*i*_) for Fisher’s exact test.

	*C*_*today*_	*C*_*history*_
*w*_*i*_=1	# today tweets containing *w*_*i*_ *,* (*k*)^a^	# history tweets containing *w*_*i*_ *(K)* ^b^
*w*_*i*_=0	# today tweets not mentioning *w*_*i*_ *, (n-k)*	# history tweets not mentioning *w*_*i*_ *(N-K)*
	*n* ^c^	*N* ^d^

^a^ *k:* the number of tweets containing the keyword *w*_*i*_ today.

^b^ *K:* the number of times the keyword *w*_*i*_ was mentioned in the period before the festival.

^c^ *n:* the number of tweets today.

^d^ *N:* the number of tweets in the period before the festival.

## Results

As noted above, the target population was defined as any user who tweeted a hashtag related to the event during the data period. To validate this heuristic, a random sample of 200 twitter users who mentioned the Wakestock festival in their tweets were analyzed. Their tweets were labeled as to whether or not the tweets of a user implied that they were at the event. The area under the receiver operating characteristic (ROC) curve for this label as a function of the number of tweets a user made that had the event hashtag was 0.91 and the true detection rate at the threshold of two tweets was 0.70. Therefore, the majority of people who were detected by our heuristic did, in fact, attend the festival. The remaining users either did not attend the event, and thus added noise to our analysis, or did not mention their attendance in their tweets.

Table 4 shows the list of statistically significant symptoms (at *P*<.01) identified in the Twitter data for each of the 10 events. Several observations are in order. First, though most identified symptoms are mild (eg, tired), in some events, the symptoms could be a cause for concern. For example, in the Bestival event, the symptom was “tremor”.

In only two of the events (Wakestock and V Festival) did all three methods identify the same symptoms. Anecdotally, once “cough” was identified as a possible symptom after the Wakestock festival, we found tweets such as “anyone else still suffering from the wakestock cough? can’t be only me”, which were made by people who were identified as having been to the festival, suggesting that this is a true symptom that was also self-identified as due to the event. This, together with the fact that it was identified by all three analysis methods, indicates that this symptom is very unlikely to be a spurious false positive, especially as it was identified by making different comparisons within the data (eg, target vs control population and before vs after the event in the target population). Thus, the use of more than one analysis method strengthens the analysis and reduces the likelihood of false positives.

We tested the agreement between all pairs of analysis methods for each of the events using a chi-square test at a threshold of *P*=.01. Methods 2 and 3 had a statistically significant agreement in six of the 10 events, Methods 1 and 3 in two of eight events (two of the events had no identified symptoms), and Methods 1 and 2 in three of eight of the events. We also found a statistically significant agreement between sources for three of the events: Wakestock, V Festival, and T in the Park. The agreement rate expected by chance, as computed using an FDR procedure, is 5 of 1000 comparisons. Therefore, these agreements are much higher than expected by chance and lend support to the hypothesis that the different methods identified real signals, through alternative means.

Table 5 shows the list of statistically significant symptoms (at *P*<.01) identified in the Bing data for each of the 10 events using Method 2. We applied only this method because there was insufficient daily activity in the Bing data to allow the application of Methods 1 and 3. As this table shows, the symptoms identified in the Bing data were potentially more serious (eg, “diarrhea” and “vomiting”) and also more personally sensitive. This is probably because users tend to share more sensitive information in anonymous media [46]. Thus, the use of Bing data complements Twitter data in the kinds of symptoms that are identified. However, the relative sparseness of this data, which is at least partly related to the number of Bing users in the United Kingdom, also means that not all methods are applicable to it.

In order to validate whether our methods might result in false positive symptoms, we also applied our methods to an event with a small physical footprint, but one that had significant media attention. Specifically, we chose the opening of The Shard building in London (the tallest building in the European Union) on July 5, 2012. This event was mentioned by 2007 users in 5553 tweets. No symptoms were reported at statistically significant levels by any of these methods. This provides evidence that when no symptoms exist, our methods will not report spurious symptoms.

**Table 4:** Statistically significant symptoms^a^ from Twitter data for each event and three analysis methods.

Event	Method 1	Method 2	Method 3
Wakestock	Cough	Cough	Tired, cough
Wireless Festival	None	Tired, pain, tremor	Tired, flatulence
T in the Park	Tired	Tired, pain, cough	Tired, cough
V Festival	Depression	Tired, pain, depression	Depression
Bestival	None	Tired, pain, tremor	Tired, fever
Creamfields	None	Tired, pain, blindness	None
Hajj	Rash, wound	Tired	Tired
Isle of Wight Festival	None	Bleeding	None
Download Festival	None	None	None
RockNess	None	Phobia, swelling	None

^a^When more than three symptoms were significant, only the top three are shown.

**Table 5:** Statistically significant symptoms^a^ from Bing data for each event using Method 2.

Event	Method 2
Wakestock	Pain
Wireless Festival	Pain
T in the Park	Wound, cough, diarrhea
V Festival	Perspiration, edema, wound
Bestival	Vomiting, diarrhea
Creamfields	Wound, rash, itch
Hajj	Fever, flatulence, pain
Isle of Wight Festival	Headache, fever, flatulence
Download Festival	Diarrhea, wound, headache
RockNess	Fever

^a^When more than three symptoms were significant, only the top three are shown.

## Discussion

### Principal Findings

Mass gatherings are potentially significant to the spread of infectious diseases. However, traditional surveillance methods are challenged by the fact the participants may congregate and disperse very quickly. In this paper, we investigated whether syndromic surveillance based on Twitter and query logs could be used to monitor mass gatherings.

We looked at nine music festivals that took place in the United Kingdom in 2012 as well as the 2012 Hajj religious gathering in Mecca. When analyzing the Twitter data, we considered three different statistical methods. The three methods did not always give the same results, with Methods 1 and 3 finding no statistically significant symptoms almost half of the time. However, when all three methods did identify statistically significant symptoms at the same concert, there was almost always agreement with at least one of the symptoms.

Each of the three methods compares different attributes of the data in order to detect medical symptoms. Because of this, each method might be better in the analysis of data from some festivals, while for others it will perform less accurately. By using more than one method, we afford two benefits. First, if more than one method discovers a symptom has appeared with an unexpectedly high probability (as noted above), this strengthens the evidence that this symptom has indeed appeared in festival participants. Second, at the cost of higher false positive rates (but also higher true positives), health authorities might choose to use symptoms discovered by any of the methods as possible candidates for further investigation.

The relative lack of data provided by the Bing query logs permitted only Method 2 to be used. Generally, the statistically significant symptoms that were identified were different from the symptoms identified by Twitter. We hypothesized that this is because users rightly perceive that tweets are public, while queries are private. Consequently, the symptoms identified by the query log describe more private indicators such as “flatulence” and “diarrhea”. Nevertheless, for two concerts, namely “Wirelessfest” and “T in the Park”, using Method 2 for both Tweets and query logs, the same symptoms were identified as “pain” and “cough” respectively.

### Limitations and Conclusions

To the best of our knowledge, no infectious outbreaks at mass gatherings were reported to health authorities during the last 18 months, the period for which query logs are available. While this is, of course, fortunate, it prevents any comparison with ground truth data. Future work is needed to compare results from Internet data with results obtained from traditional methods. Note, however, that the use of traditional surveillance methods can be challenging in the context of mass gatherings due to the combination of an incubation period prior to onset of symptoms and dispersal of participants to their home regions.

An additional drawback of our method is that some of the identified symptoms (eg, tired) might not be a symptom of a disease, but instead the outcomes of going to specific types of events. Therefore, an additional filtering stage might be required so as to remove symptoms that regularly appear in similar events.

## Multimedia Appendix 1

Number of tweets containing symptom words for each festival.

## Abbreviations

CDC: Centers for Disease Control and Prevention
EMS: Emergency Medical Services
FDR: false discovery rate
ILI: influenza-like illness
OTC: over the counter
WHO: World Health Organization
