# Supplementary material for: Detecting Disease Outbreaks in Mass Gatherings Using Internet Data
Source: J Med Internet Res. 2014 Jun 18;16(6):e154. doi: 10.2196/jmir.3156 (PMC4090384; doi:10.2196/jmir.3156)
Supplement: Supplementary file 1 [file jmir_v16i6e154_app1.pdf]

Multimedia Appendix 1: Number of tweets containing symptom words for each festival

The table below is a count of the number of tweets which contained a symptom word or its synonyms in each of the festivals analyzed. Only symptoms which had a total of 10 or more appearances are listed.

	Festival									
**Symptom**	**Wakestock**	**Wireless**	**Tinthepark**	**VFestival**	**Bestival**	**Creamfields**	**Hajj**	**Isleofwightfest**	**Download**	**Rockness**
**Agoraphobia**	0	5	8	7	11	7	5	0	0	0
**Alexia**	0	36	20	17	76	12	12	1	0	2
**Alopecia**	1	17	22	23	14	28	5	0	0	0
**Amnesia**	1449	64	64	471	509	3226	63	3	3	13
**Anorexia**	59	105	107	201	392	336	100	3	2	14
**Anxiety**	67	98	111	475	441	391	118	5	7	11
**Apnea**	0	69	69	462	8560	874	89	0	6	7
**Arachnophobia**	2	12	26	55	17	36	8	0	0	0
**Arrhythmia**	0	0	1	1	2	1	7	0	0	0
**Ataxia**	0	0	4	0	10	2	4	0	0	0
**Back ache**	4	7	9	14	9	13	28	0	0	0
**Belching**	90	73	86	360	296	354	79	3	2	10
**Bleeding**	268	68	74	1194	812	1245	98	10	35	25
**Blindness**	558	66	70	4558	3119	3292	83	25	26	80
**Blister**	175	64	65	770	311	621	63	2	10	24
**Bloating**	39	64	60	143	96	256	62	0	2	5
**Bruise**	465	71	67	1710	805	1357	74	15	10	25
**Childbirth**	303	109	108	1355	1925	2573	113	26	21	39
**Chorea**	0	0	0	1	2	4	4	0	0	0
**Claustrophobia**	12	50	53	129	32	51	48	1	0	1

**Constipation**	27	58	63	165	84	144	65	2	1	1
**Convulsion**	0	13	29	89	14	8	45	0	1	0
**Cough**	665	75	79	3122	1526	2357	78	12	24	51
**Cramp**	227	65	64	696	451	835	65	5	4	13
**Deformity**	37	60	58	117	99	200	61	0	1	0
**Depression**	2739	74	67	8586	4696	11244	71	58	67	144
**Diarrhea**	37	92	111	110	103	159	109	0	1	6
**Dizziness**	308	78	72	436	296	678	88	4	12	12
**Dyspepsia**	11	28	35	28	31	33	47	0	0	0
**Edema**	0	4	2	1	0	12	9	0	0	0
**Epistaxis**	24	46	60	89	46	80	34	0	0	3
**Euphoria**	31	61	66	185	315	342	70	3	3	8
**Fever**	364	65	64	569	933	1040	71	17	13	31
**Flatulence**	570	77	106	2114	1447	3256	82	15	40	39
**Hallucination**	17	54	58	245	160	153	64	1	0	0
**Headache**	683	64	65	3419	1383	2720	65	15	27	26
**Hirsutism**	0	0	6	0	7	2	12	0	0	0
**Hyperthermia**	0	16	26	31	12	35	6	0	0	0
**Hyperventilation**	17	61	58	349	148	49	36	0	0	0
**Hypothermia**	15	19	41	22	22	38	27	1	0	1
**Impotence**	2	14	24	28	35	19	59	0	0	11
**Infertility**	10	16	23	12	213	16	61	7	0	1
**Insomnia**	135	68	69	499	585	1079	97	3	3	7
**Itch**	110	65	64	717	278	586	67	8	15	13
**Jaundice**	3	7	16	8	13	12	23	0	0	2
**Malaise**	0	9	17	4	46	4	35	0	0	0
**Melena**	0	4	3	11	42	34	2	0	0	1
**Miscarriage**	4	34	33	24	41	50	55	1	0	0
**Nausea**	1	31	31	27	75	53	60	0	0	0
**Nystagmus**	21	0	0	0	0	9	3	0	0	0

**Pain**	2231	87	79	11368	6941	11006	99	86	122	177
**Palpitation**	2	48	45	48	80	81	45	1	0	2
**Paralysis**	10	64	76	75	59	123	111	3	2	7
**Paranoia**	263	125	135	847	764	1024	129	8	27	23
**Persecution**	1	24	30	44	275	95	69	1	0	3
**Perspiration**	637	96	105	4184	1975	4682	116	21	27	53
**Phobia**	62	82	81	366	259	377	114	2	5	10
**Rash**	66	65	64	227	271	408	65	3	3	2
**Rigor**	2	10	25	44	52	106	54	1	1	2
**Shivering**	138	64	64	950	454	1074	66	7	3	14
**Sleepy**	658	65	70	3968	1729	2964	65	16	32	52
**Somnolence**	25	46	49	35	40	48	67	0	0	0
**Swelling**	91	64	64	275	254	379	65	3	3	10
**Tachycardia**	0	3	2	0	1	0	11	0	0	0
**Thirst**	20	66	64	662	139	216	67	22	24	29
**Thirsty**	133	64	64	915	406	985	66	6	4	10
**Tinnitus**	2	18	48	19	57	66	13	0	0	0
**Tired**	5009	143	137	24094	9624	19008	107	145	158	282
**Toothache**	40	61	64	169	64	242	61	0	2	1
**Tremor**	809	86	91	5172	4462	5410	120	46	56	139
**Urticaria**	10	55	64	162	578	263	57	0	12	19
**Vertigo**	13	25	44	50	329	174	32	0	0	2
**Vomit**	197	64	71	904	1107	1059	66	14	13	13
**Weak**	351	73	71	3318	1774	2291	82	15	30	70
**Weight loss**	4	19	26	121	119	45	56	0	1	2
**Wound**	239	72	71	898	637	1975	82	21	12	55
